# Effect of porous density of twisted tape inserts on heat transfer performance inside a closed conduit

**DOI:** 10.1016/j.heliyon.2023.e21206

**Published:** 2023-10-24

**Authors:** Md Tauhidur Rahman, Khairul Habib, Md Niamul Quader, Navid Aslfattahi, Kumaran Kadirgama, Likhan Das

**Affiliations:** a Department of Petroleum and Mining Engineering, Military Institute of Science and Technology, Mirpur Cantonment, Dhaka, 1216, Bangladesh; b Department of Mechanical Engineering, Universiti Teknologi PETRONAS, Perak, Malaysia; c Department of Electrical Engineering and Computer Science, South Dakota School of Mining & Technology, Rapid City, SD, USA; d Department of Fluid Mechanics and Thermodynamics, Faculty of Mechanical Engineering, Czech Technical University in Prague, Technická 4, 166 07, Prague, Czech Republic; e Faculty of Mechanical and Automotive Engineering Technology, Universiti Malaysia Pahang, Pekan, 26600, Malaysia; f Almaaqal University, College of Engineering, Department of Civil Engineering, Basra, 61003, Iraq; g Department of Industrial and Manufacturing Systems Engineering, Iowa State University, 515 Morrill Road, Iowa, Ames, 50011, USA

**Keywords:** Heat transfer, Porous density, Twisted tape insert, Heat exchangers, Reynolds number

## Abstract

This study examines the impact of varying the porosity density of twisted tape inserts (TTI) on the temperature distribution, fluid velocities, heat transfer coefficients (HTC), Nusselt numbers (*Nu*), turbulent kinetic energy (TKE), and performance from 5000 to 12500 Reynolds numbers (*Re*). The entire process involved the design of TTIs and double pipe heat exchangers using SolidWorks. Subsequently, a three-dimensional fluid flow model was employed to solve equations related to energy mass, energy, and momentum within the ANSYS Fluent interfaces. The findings highlight the noteworthy impact of high porosity TTIs, which consistently reduce temperature spans, increase fluid velocities, and greatly HTC and *Nu* when compared to low porosity TTI, typical TTI, and plain tubes. Furthermore, high porosity TTI significantly increases TKE, indicating increased fluid turbulence and higher heat transfer efficiency, especially at Re = 12500. The assessment of PEC emphasizes the superiority of high porosity TTI, demonstrating their significant performance increase potential of over 6.44 % over low porosity TTI and a staggering 62.5 % above typical TTI. In conclusion, high porosity TTI emerges as a potential solution for improving heat transfer efficiency and overall system performance in a variety of industrial applications, promising enhanced energy efficiency and superior performance.

## 1 Introduction

Heat exchangers are devices that transfer heat from two or more dissimilar fluids, such as liquids, vapors, or gases, to one another. The heat transfer process can be gas-to-gas, liquid-to-gas, or liquid-to-liquid, and it can occur through a solid separator, which keeps the fluids from mixing, or direct fluid contact, regardless of the type of heat exchanger utilized [[1], [2], [3], [4]]. There are a lot of technical materials on heat exchanger design, operation, and maintenance [5]. During the construction of a heat exchanger system, a variety of parameters, such as the material of the shell and tube, the structural design, and the properties of the fluid that will flow through the tube, are important in conveying liquid or gaseous fluids effectively and efficiently. As a result, heat exchanger design and systems are crucially important, especially in plants and industry.

There are numerous ways to expand heat exchanger heat transfer effectiveness [[6], [7], [8]]. As a rule, strategies for further developing heat transfer can be partitioned into three general classes: active, passive, and combined methods. Active and passive heat transfer enhancement techniques are widely employed in various applications. Active heat transfer enhancement necessitates the presence of external energy, such as magnetic field vibrations. On the other hand, passive heat transfer enhancement does not require any additional effort but results in increased energy loss from the flow.

A lot of approaches have been utilized for the enhancement of heat transfer efficiency by several researchers. Heat exchanger tubes and other thermal systems frequently utilize TTIs to boost heat transfer [[9], [10], [11], [12]]. Kumar et al. (2018) conducted an experimental study to determine the convective heat transfer, friction factor, efficacy, and number of transfer units (NTU) of Fe3O4/water nanofluid flow in a twin pipe U-bend heat exchanger with temperature measuring instruments (TTIs) [9]. They employed TTIs with H/D = 10, 15, and 20 particle volume concentrations ranging from 0.005% to 0.06% and Reynolds numbers (*Re*) ranging from 16,000 to 32,000 in their studies. At 0.06% concertation of nanofluids, the *Nu* increased to 14.76%, while with a TTI twist ratio (H/D) of 10 included, the *Nu* increased to 38.75%. When comparing the data for water, it was observed that the friction factor was 1.092 times higher without the use of TTIs and 1.251 times higher with the use of TTIs. These observations were made at a twist ratio (H/D) of 10 and a Re of 30,000. Nakhchi and Esfahani (2021) used heat exchangers augmented with double V-cut TTIs to investigate the turbulent features and thermal enhancement parameter of CuO–water nanofluids [13]. The two-fold V- cut TTIs increase the *Nu* of the nanofluid flow. Tiwari et al. (2021) focused their research on investigating the impact of various thermal factors, including total heat transfer and efficacy [14]. They employed WO_3_/water nanofluid with diverse, unique TTIs, rib, and porous plate. The utilization of a rib-type inserts in both experimental and computational fluid dynamics methodologies resulted in achieving the highest heat transfer rates of 1767.91 Wm^−2^K^−1^ and 1702.71 Wm^−2^K^−1^, along with corresponding efficacies of 1.86 and 1.79. These outcomes were obtained when employing a 1% optimum volume concentration of WO3/water nanofluid. For both experimental and computational fluid dynamics approaches, utilizing a rib-type insert improved total heat transfer and effectiveness by 11.84 %, 12.38 % 14.56 %, 14.30 %, respectively. In their study, Das et al. (2021) made modifications to the conventional TTI by incorporating various geometrical cuts (triangular, rectangular, and circular) on its surface. Additionally, they employed a novel class of surfactant-free Ionanofluid in their experiments [15]. They discovered that the rectangular-cut TTI demonstrates the greatest improvement in heat transfer, as the additional vortices generated by these particular cut-inserts have a stronger impact compared to other alternatives.

In recent years, a lot of research to understand the improvement of heat transfer is being done. Table 1 presents a comprehensive overview of the studies conducted on the enhancement of heat transfer. None of the studies clearly explain the effect of the porous density of the twisted tape from low density to high density. In addition, no specific analysis is performed on the optimum drilling ratio of the twisted tape between the heat transfer coefficient and the pressure drop (friction loss). Apart from that, most publications describe the characteristics of traditional twist tape inserts and the various twist ratios of common TTIs. There is no specific mention of the porosity and density of TTIs.

**Table 1:** Dimensional parameters of the designed pipe.

Parameter	Length (mm)	Diameter (mm)	Thickness (mm)
**Inner Tube**	1200	25.4	1
**Outer Tube**	1000	50.8	2
**Insert**	1200	N/A	1

This study aims to fill a substantial knowledge gap by performing a thorough assessment of the complicated impacts of modifying the density of pores within TTIs, which are used to increase heat transfer in narrow spaces. The geometry of these inserts has received the majority of attention in earlier studies, with the importance of pore density in affecting heat transfer efficiency being largely overlooked. This research seeks to identify the varied ways in which these differences affect the heat transfer process through a systematic exploration covering a range of pore densities, from low to high. These porous TTIs will be compared to conventional TTI designs as well as plain tubes in order to analyze how well they function thermally. As a result of the new perspectives on fluid dynamics, the findings from this research have the potential to change the way we construct heat exchangers.

## 2 Methodology

### 2.1 Heat exchanger and perforated twisted tape model

Dimension references on the ASME (American Society of Mechanical Engineers) Code and TEMA (Tubular Exchangers Manufacturers Association).

● Double pipe sections have been designed for up to 165 bar (2400 psig) on the shell side and up to 1033 bar (15,000 psig) on the tube side. Metal-to-metal ground joints, ring joints, or confined O-rings are used in the front-end closures at lower pressures.
● Commercially available single-tube double-pipe sections range from 50.8 through 101.6 mm (2 through 4-inch) pipe size shells, with inner tubes varying from 19 to 63.5 mm (3⁄4 to 21⁄2 inch) pipe size. These can be justified economically if the equivalent shell and surface required are less than 27.8 m^2^ (300 ft^2^).

**Fig. 1:**
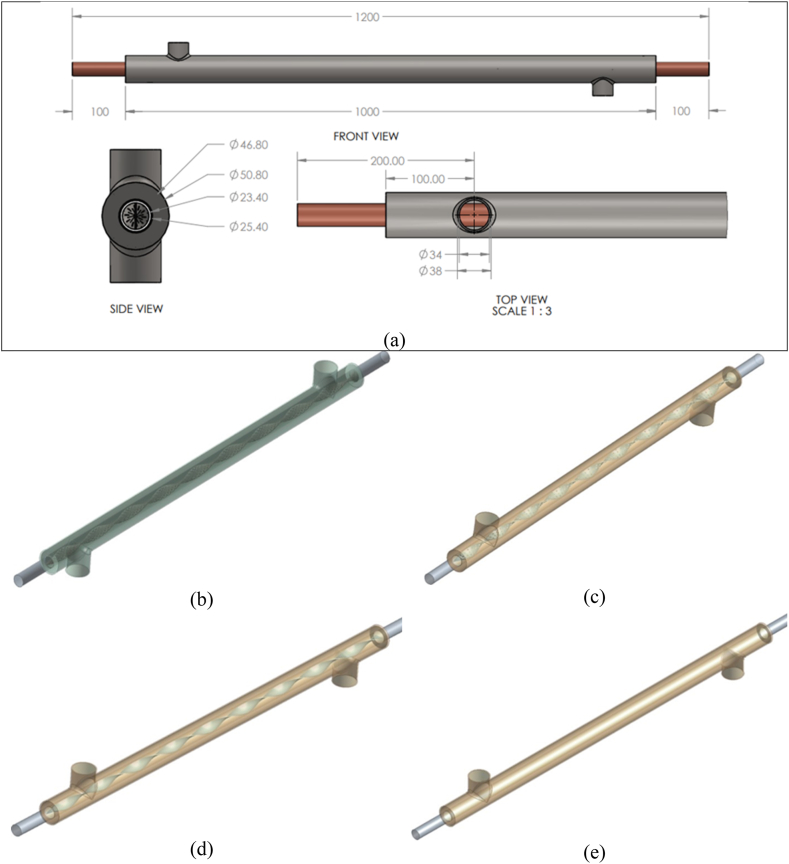
(a) Detail dimensions of the double pipe heat exchanger, (b) Double pipe heat exchanger with high porosity inserts geometry model, (c) Double pipe heat exchanger with low porosity inserts geometry model, (d) Double pipe heat exchanger with regular insert geometry model, (e) Double pipe heat exchanger without insert geometry model.

Based on the specifications in references, the double pipe heat exchanger is constructed and designed using SolidWorks, solid modeling, and computer-aided design. The design parameters are shown in Table 1. The TTIs were used both conventionally and perforated to compare and evaluate the hydrothermal performance of the heat exchanger. Fig. 1(a-e) represents the detailed dimensions and geometry model of high, low, regular, and without porosity inserts of double pipe heat exchangers. Ansys-Fluent simulation interface was incorporated to define and solve the problem within a defined boundary.

### 2.2 Assumptions, boundary conditions, governing equations and data reduction

The current investigation runs under the assumption that the fluid flow under examination exhibits characteristics of turbulence, incompressibility, and a state of steady equilibrium. The selection of turbulent flow is based on its inherent characteristics of chaotic mixing, which result in enhanced heat transfer rates and overall efficiency of heat exchange processes. Complete thermal development was achieved by considering an appropriate length to account for fully developed flow and heat transfer mechanisms. This allowed for a uniform temperature distribution and the maintenance of a constant downstream temperature. By accomplishing a steady-state flow, enables efficient heat transmission across the fluid and equilibrates temperature differences for full thermal development. Nevertheless, the current analysis does not take into account the dissipation of energy caused by the movement of fluid. The wall of the pipes and inserts were considered as non-slip boundary conditions. The phenomenon of fluid flow interacting with solid walls is characterized by thermal coupling.

A velocity inlet condition is implemented at the inlet of both pipes to initiate the flow of fluid. On the other hand, a zero-pressure outlet condition is employed at the exits of both the tube and annulus sides. The computational methodology employed in this study fails to consider the effects of viscous dissipation. The fluid on the hot side of the system is introduced at a temperature of 353 K, while the fluid on the cold side enters at a temperature of 298 K. The study investigated the effects of different flow velocities on the inner tube fluids within a range of Reynolds numbers from 5000 to 12500 while the flow rate was constant at Re = 12500 at the outer tube.

Taking the above-mentioned assumptions and boundary conditions into consideration, the fluid flow as well as heat transfer characteristics are defined with differential governing equations. The continuity, energy, and momentum equations that are employed for the three-dimensional model are expressed below:

Equation (1) represents the continuity equation.

(1) $$\frac{\partial }{\partial x_{i}}\left(\rho u_{i}\right)=0$$

Momentum equations can be written as Equation (2).

(2) $$\frac{\partial \left(\rho u_{i}u_{j}\right)}{\partial x_{j}}=-\frac{\partial p}{\partial x_{i}}+\frac{\partial }{\partial x_{j}}\left[\mu \left(\frac{\partial u_{i}}{\partial x_{j}}+\frac{\partial u_{j}}{\partial x_{i}}-\frac{2}{3}\delta_{ij}\frac{\partial u_{k}}{\partial x_{k}}\right)\right]+\frac{\partial }{\partial x_{j}}\left(-\rho \underline{{u´}_{i}u_{j}´}\right)$$

Here, $-\rho \underline{{u´}_{i}u_{j}´}$ is termed as Reynolds stresses, which can be modeled by Boussinesq hypothesis that relates $-\rho \underline{{u´}_{i}u_{j}´}$ to the mean velocity gradient expressed in Equation (3).

(3) $$-\rho \underline{{u´}_{i}u_{j}´}=\mu_{t}\left(\frac{\partial u_{i}}{\partial x_{j}}+\frac{\partial u_{j}}{\partial x_{i}}\right)-\frac{2}{3}\left(\rho k+u_{t}\frac{\partial u_{k}}{\partial x_{k}}\right)\delta_{ij}$$

Here, $u_{t}$ is the turbulent viscosity, which can be expressed as Equation (4).

(4) $$\mu_{t}=\rho C_{\mu }\frac{k^{2}}{\varepsilon }$$

Equation (5) and Equation (6) represent the energy equation.

(5) $$\frac{\partial }{\partial x_{i}}\left[u_{i}\left(\rho E+P\right)\right]=\frac{\partial }{\partial x_{j}}\left(k_{eff}\frac{\partial T}{\partial x_{j}}\right)$$

(6) $$E=h-\frac{P}{\rho }+\frac{u^{2}}{2}$$

To ensure the accuracy of predictions for dynamics of flow, this model is further evaluated by some turbulence models, including standard k-ω model, standard k-ε model, Renormalized Group (RNG) k–ε model, and shear Stress Transport (SST) k–ω turbulence model.

In the field of fluid dynamics, the parameter *Nu* holds significant importance as it represents the ratio of convective and conductive heat transfer at a boundary within a fluid flow regime. This parameter can be determined by utilizing Equation (7).

(7) $$Nu=\frac{1}{A}\int {Nu}_{x}dA$$

Where ${Nu}_{x}$ is the local *Nu* that can be expressed by Equation (8).

(8) $${Nu}_{x}=\frac{h_{x}d_{h}}{k}$$

Where, $h_{x}$ is the local HTC*,* $d_{h}$ is the equivalent hydraulic diameter and k expresses the thermal conductivity of the working fluid.

For diameter will be using the hydraulic diameter, $d=d_{h}$, The friction factor (f) is another significant parameter that has a notable impact on the friction loss, heat transfer rate, and effectiveness of the heat exchanger. The Darcy-Weisbach equation is utilized to determine the friction factor (f) based on the pressure drop (ΔP) occurring along the length (L) of a tube, as represented by Equation (9).

(9) $$f=\frac{\Delta P}{\frac{L}{D}\left(\rho \frac{V^{2}}{2}\right)}$$

The computation of the performance evaluation coefficient (PEC) was conducted in order to examine the thermal performance of the corrugated channel. This evaluation took into account the enhancement ratio of the *Nu* and the frictional loss, as specified in Equation (10).

(10) $$PEC=\frac{\frac{Nu}{{Nu}_{plain\;tube}}}{{\left(\frac{f}{f_{plain\;tube}}\right)}^{\frac{1}{3}}}$$

### 2.3 Mesh study of model using ANSYS

**Fig. 2:**
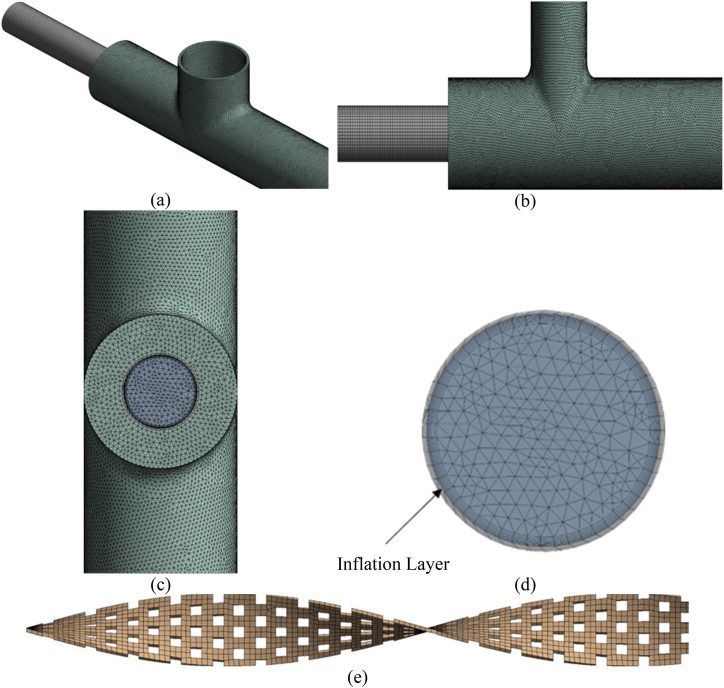
Meshing of the different solid and fluid domains. (a) Isomeric view, (b) Front view, (c) Right side view, (d) Inner tube, fluid domain and inflation layer, (e) perforated twisted tape.

ANSYS Fluent pre-processing gear has been used to construct the mesh on every of the area geometry. For all sixteen situations, a finer mesh has been used to generate results with high-quality precision. In order to consider the impact of the boundary layer, an inflation layer was implemented on the inner tube of the double pipe heat exchanger. This inflation layer was designed to achieve an equivalent Y+ value of 1, with a growth rate of 1.2. The mesh was generated to the finest level. An inflated layer on the tube wall was introduced to predict the behavior of a thick boundary layer. This allowed simulation and investigation of thick boundary layer features and effects, including velocity, temperature, and pressure gradients near the wall. This computational fluid dynamics method for researching boundary layer behavior is common and effective. After generating the mesh define all the domain accordingly to the geometry. Fig. 2(a-e) displays meshes for double pipe heat exchanger with high porosity TTI geometry. The grid sensitivity test is presented in Table 2 which shows that the average outlet temperature become converged at an elements number of 2.8 × 10^6^ with a negligible error of 0.38 %.

**Table 2:** Grid sensitivity test for conventional inserted tube.

Elements No	Re	Average Outlet temperature (K)	% of error
0.8 × 10^6^	10000	301.8	
1.5 × 10^6^		326.5	7.75
2.1 × 10^6^		335.6	2.8
2.5 × 10^6^		339.5	1.2
2.8 × 10^6^		340.8	0.38
2.9 × 10^6^		340.2	0.2

## 3 Results and discussion

### 3.1 Simulation validation

This study evaluated the performance of the turbulence model using a plain tube as the initial reference. The objective was to assess the accuracy and reliability of the model when used with tube heat exchanger that includes various TTIs. To validate the accuracy of the simulations, a comparison was conducted between the *Nu* from the simulation and that from Gnielinski's equation, which is a widely recognized reference. The findings demonstrated a strong correlation between the simulations and Gnielinski's equation, with deviations of less than 10 %. These findings indicate a satisfactory level of accuracy in the simulations. Table 3 illustrates comprehensive validation data.

**Table 3:** Validation of Simulation Results with Gnielinski's equation for *Nu.*

Reynold's Number, *Re*	Friction factor, *f*	Nusselt number, *Nu*	Nusselt Number (Gnielinski's equation), *Nu*_*Gniel*_	Error (%)
5000	0.033	33.15	34.67	4.38
12500		44.45	45.62	2.57

### 3.2 Effect of various design inserts on temperature, velocity, and pressure

**Fig. 3:**
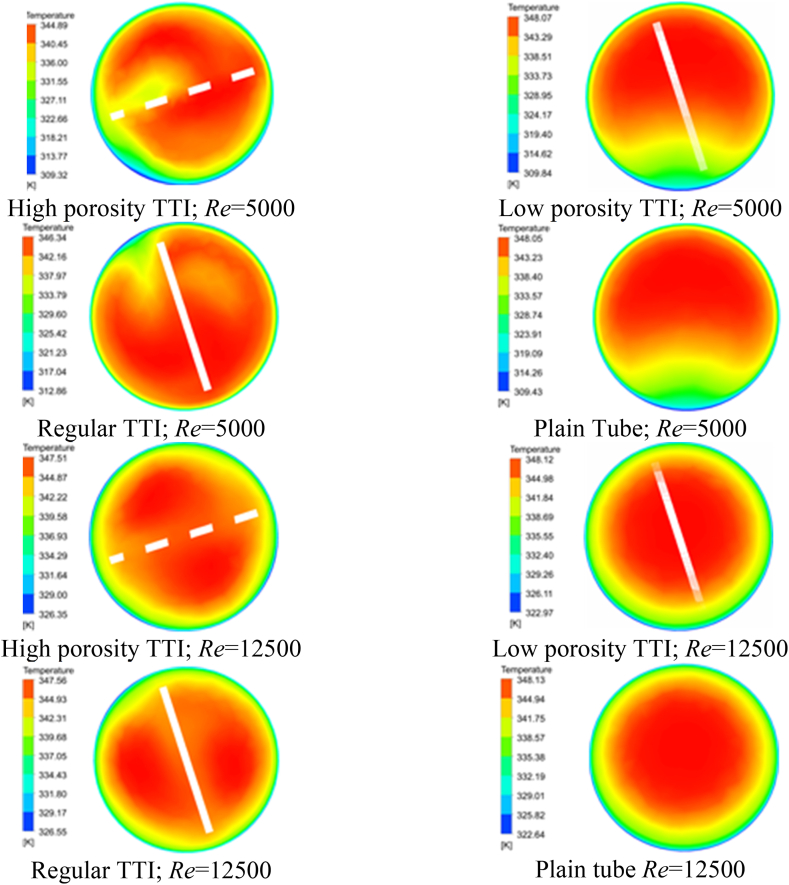
Temperature Contour (Midplane). (a) at Re 5000; flow condition: high porosity TTI, (b) at Re 5000; flow condition: low porosity TTI, (c) at Re 5000; flow condition: regular TTI, (d) at Re 5000; flow condition: plain tube, (e) at Re 12500; flow condition: high porosity TTI, (f) at Re 12500; flow condition: low porosity TTI, (g) at Re 12500; flow condition: regular TTI, and (h) at Re 12500; flow condition: plain tube.

The distribution of temperature and pressure was affected by the dynamics of the flow and the properties of the fluid. The use of TTIs in conduit architecture resulted in increased circumferential velocity and improved fluid mixing. Consequently, a more efficient heat transfer occurred between the central and peripheral fluid zones. This phenomenon is clearly explained through the visual representation in Fig. 3(a-h) and supported by the detailed tabulation in Table 4. These depictions focus on the midplane temperature contours, specifically considering Re of 5000 and 12500.

**Table 4:** Temperature range of the midplane contour for different flow conditions.

*Re*	Temperature range (K)			
	High porosity TTI	Low porosity TTI	Regular TTI	Plain Tube
5000	309.32–344.89	309.84–348.07	312.86–346.34	309.43–348.08
12500	326.35–347.51	322.97–348.12	326.55–347.56	322.64–348.18

The temperature ranges were significantly affected by the specific design of the TTI. A comparative analysis of temperature ranges in different designs demonstrates that both high porosity and low porosity TTIs resulted in reduced temperature spans compared to regular inserts and plain tubes. The high-porosity TTI variant demonstrated the lowest temperature contour due to its superior thermal conductivity compared to other TTI configurations.

**Fig. 4:**
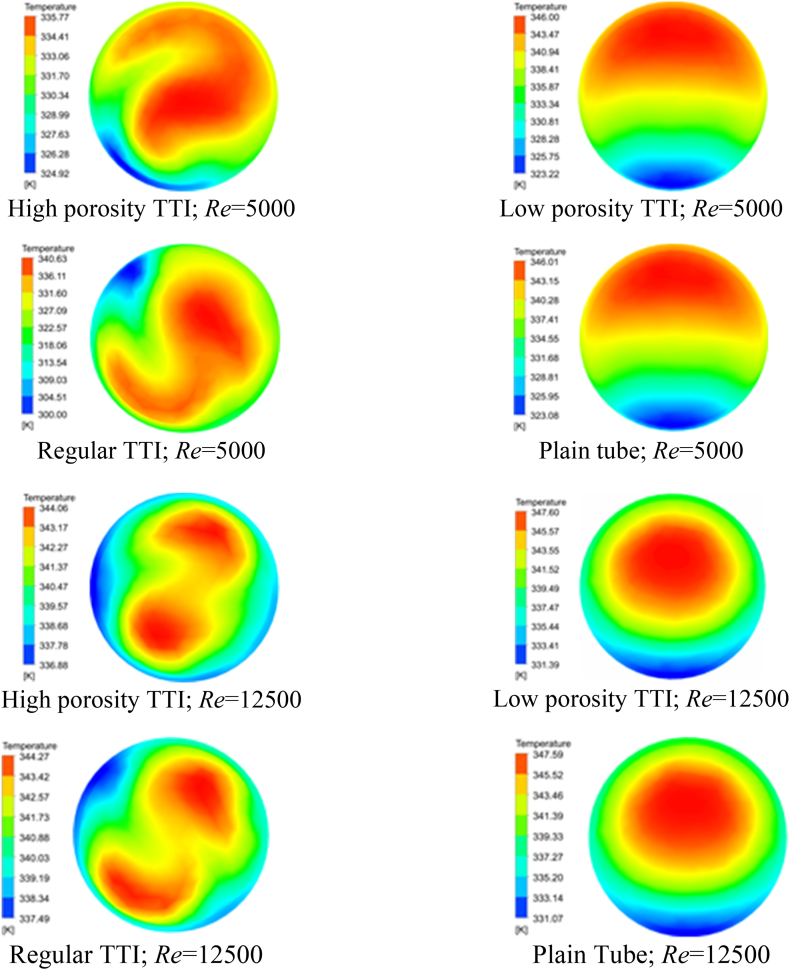
Temperature Contour (Outlet). (a) at Re 5000; flow condition: high porosity TTI, (b) at Re 5000; flow condition: low porosity TTI, (c) at Re 5000; flow condition: regular TTI, (d) at Re 5000; flow condition: plain tube, (e) at Re 12500; flow condition: high porosity TTI, (f) at Re 12500; flow condition: low porosity TTI, (g) at Re 12500; flow condition: regular TTI, and (h) at Re 12500; flow condition: plain tube.

Fig. 4(a-h) and Table 5 provides a comprehensive summary of temperature contour patterns at the outlet for different flow scenarios, specifically at Re of 5000 and 12500. Fig. 4(a-h) presents temperature ranges that illustrate the relationship between flow conditions and TTI designs. At a Re of 5000, the TTI with high porosity exhibited temperatures ranging from 324.92 K to 335.77 K, while the TTI with low porosity showed temperatures ranging from 323.22 K to 346.00 K. The regular TTI had a temperature range of 300.00 K–340.63 K, while the plain tube had temperatures ranging from 323.08 K to 346.03 K. Similarly, at Re 12500, the trends remained consistent. The high porosity TTI consistently exhibited narrower temperature ranges, suggesting a more controlled and efficient heat transfer process. The high porosity TTI is suggested as a potential method to improve heat transfer performance due to its ability to maintain a consistent temperature profile at the outlet under different flow conditions.

**Table 5:** Temperature range of the outlet contour for different flow conditions.

*Re*	Temperature range (K)			
	High porosity TTI	Low porosity TTI	Regular TTI	Plain Tube
5000	324.92–335.77	323.22–346.00	300.00–340.63	323.08–346.03
12500	336.88–344.06	331.39–347.60	337.49–344.27	331.07–347.69

**Fig. 5:**
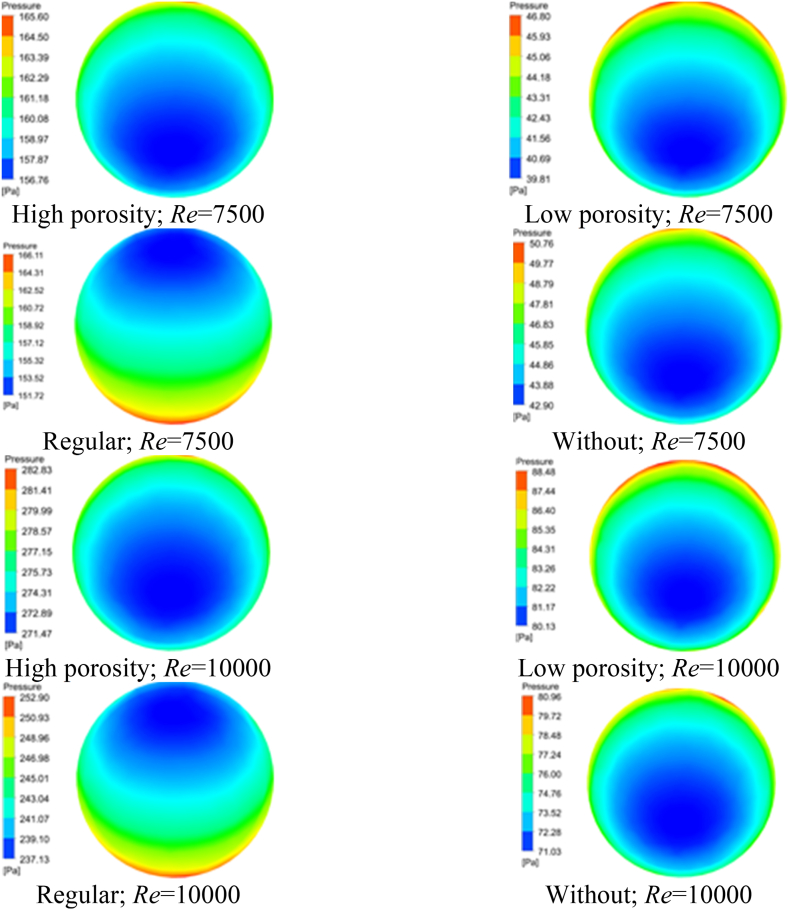
Pressure contour (inlet). (a) at Re 7500; flow condition: high porosity TTI, (b) at Re 7500; flow condition: low porosity TTI, (c) at Re 7500; flow condition: regular TTI, (d) at Re 7500; flow condition: without, (e) at Re 10000; flow condition: high porosity TTI, (f) at Re 10000; flow condition: low porosity TTI, (g) at Re 10000; flow condition: regular TTI, and (h) at Re 10000; flow condition: without.

The pressure profiles at the inlet cross-sections can be effectively analyzed by referring to Fig. 5(a-h) and Table 6. The given values pertain to the total pressure decline observed across the tube at Re of 7500 and 10000, respectively.

**Table 6:** Pressure contour at the inlet for different flow conditions.

*Re*	Inlet pressure range (Pa)			
	High porosity TTI	Low Porosity TTI	Regular TTI	Plain Tube
7500	156.76–165.60	39.81–46.80	151.72–166.11	42.90–50.76
10000	271.47–282.83	80.13–88.48	237.13–252.90	71.03–80.96

The pressure distribution observed in the plain tube configuration displayed a recurring pattern characterized by concentric zones in the shape of rings. The central zone exhibited the most minimal decline in pressure. The fluid in close proximity to the wall of the tube underwent a substantial pressure variation as a result of the development of a boundary layer. The utilization of TTI led to a rise in pressure drop. The presence of this phenomenon can be observed in the pressure-contour representation, which is believed to be caused by the surface roughness of the TTIs. The TTI with high porosity showed the highest pressure drop range. At Re 7500, the pressure drop ranged from 156.76 Pa to 165.60 Pa, and at Re 10000, it ranged from 271.47 Pa to 282.83 Pa. The pressure range significantly increased after the insertion of the TTI.

The pressure inlet contours in Table 6 represent the varying pressure conditions across different designs. At Re 7500, the TTI with high porosity had a pressure range of 156.76 Pa–165.60 Pa, while the TTI with low porosity had a range of 39.81 Pa–46.80 Pa. The regular TTI had a pressure range of 151.72 Pa–166.11 Pa, while the plain tube had pressures ranging from 42.90 Pa to 50.76 Pa. At a Re of 10000, the TTI with high porosity exhibited a range of 271.47 Pa–282.83 Pa, whereas the TTI with low porosity ranged from 80.13 Pa to 88.48 Pa. The regular TTI configuration resulted in pressure values ranging from 237.13 Pa to 252.90 Pa, while the plain tube exhibited pressures ranging from 71.03 Pa to 80.96 Pa.

In summary, the analysis of pressure profiles and pressure drops in TTI configurations and plain tubes highlights the complex relationship between flow dynamics and insert designs. The high porosity TTI is a significant factor in increased pressure drops, suggesting its ability to impact flow resistance in the conduit.

### 3.3 Effect of porosity on velocity distributions

**Fig. 6:**
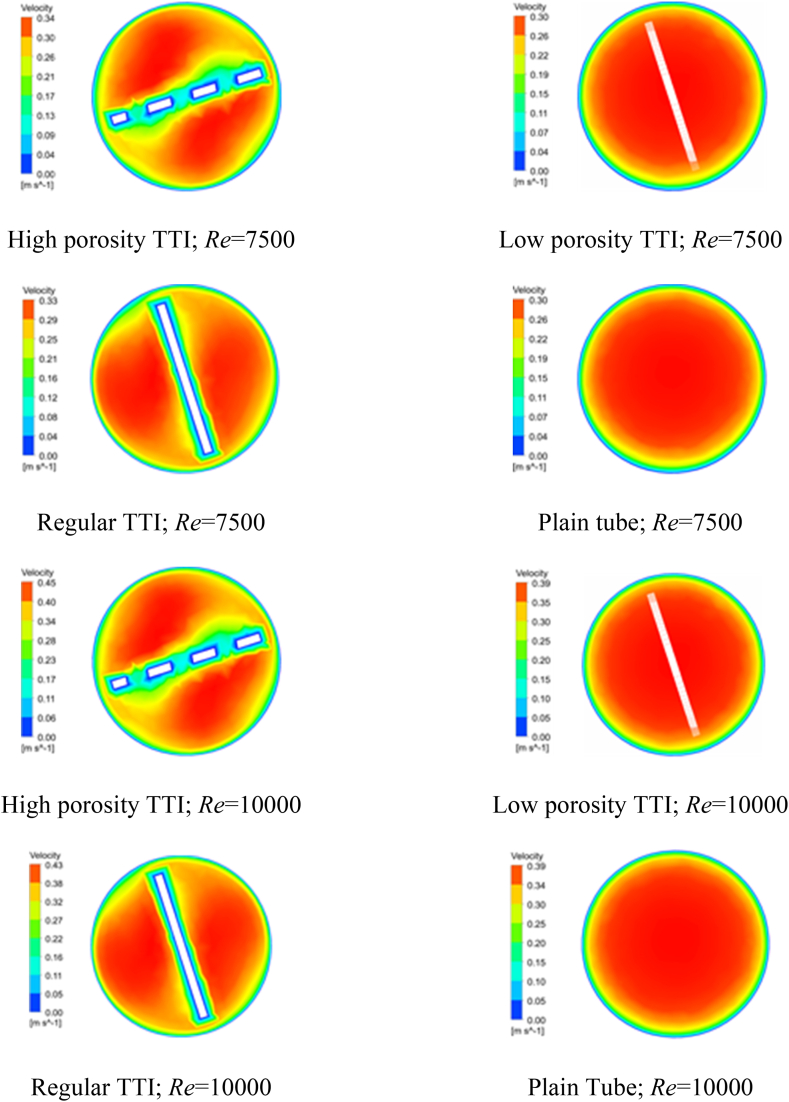
Velocity Contour at the Midplane. (a) at Re 7500; flow condition: high porosity TTI, (b) at Re 7500; flow condition: low porosity TTI, (c) at Re 7500; flow condition: regular TTI, (d) at Re 7500; flow condition: plain tube, (e) at Re 10000; flow condition: high porosity TTI, (f) at Re 10000; flow condition: low porosity TTI, (g) at Re 10000; flow condition: regular TTI, and (h) at Re 10000; flow condition: plain tube.

**Fig. 7:**
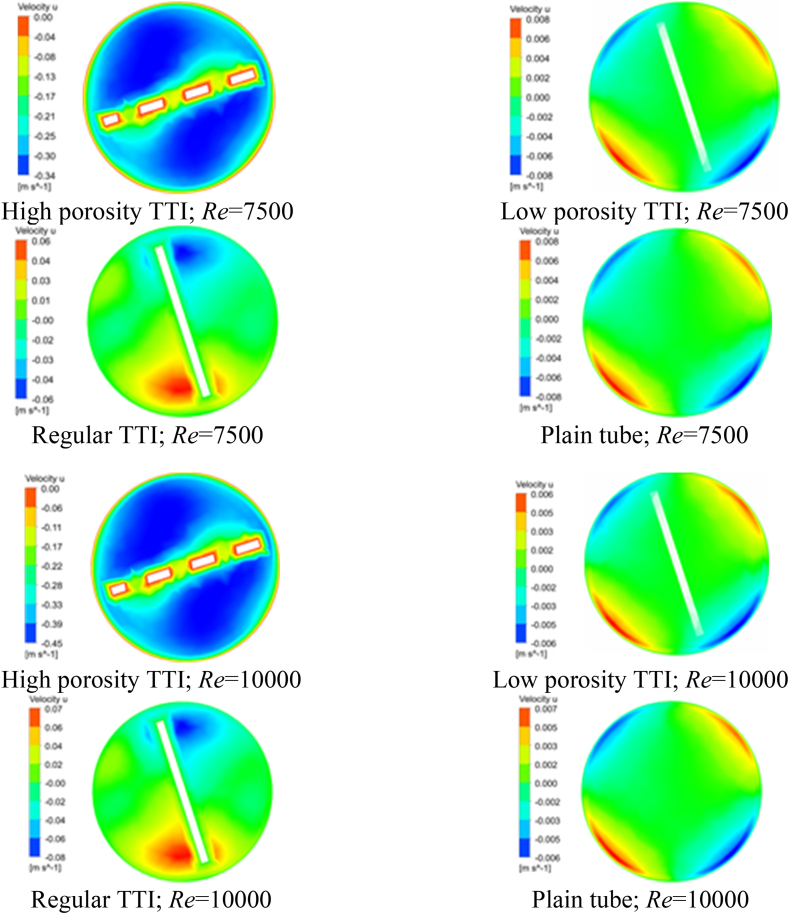
Velocity u Contour at the Midplane. (a) at Re 7500; flow condition: high porosity TTI, (b) at Re 7500; flow condition: low porosity TTI, (c) at Re 7500; flow condition: regular TTI, (d) at Re 7500; flow condition: plain tube, (e) at Re 10000; flow condition: high porosity TTI, (f) at Re 10000; flow condition: low porosity TTI, (g) at Re 10000; flow condition: regular TTI, and (h) at Re 10000; flow condition: plain tube.

**Fig. 8:**
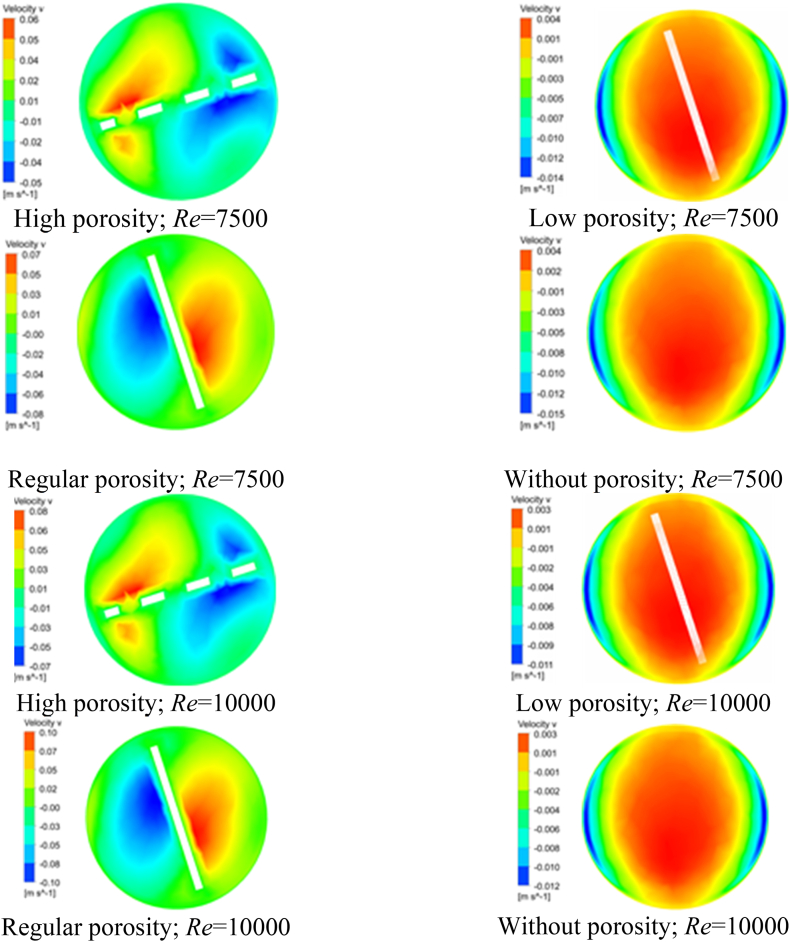
Velocity v Contour at the Midplane. (a) at Re 7500; flow condition: high porosity TTI, (b) at Re 7500; flow condition: low porosity TTI, (c) at Re 7500; flow condition: regular porosity TTI, (d) at Re 7500; flow condition: without porosity, (e) at Re 10000; flow condition: high porosity TTI, (f) at Re 10000; flow condition: low porosity TTI, (g) at Re 10000; flow condition: regular porosity TTI, and (h) at Re 10000; flow condition: without porosity.

The influence of porosity on the distribution of velocity within the heat exchanger is a crucial factor that requires thoughtful consideration. Different geometrical cuts have a significant impact on the radial and tangential velocity components, leading to noticeable consequences for TTIs with high porosity. Fig. 6(a–h), Fig. 7(a-h), and Fig. 8(a-h) illustrate the contours of axial, tangential, and radial velocities. These figures provide a detailed depiction of the behavior of water within the heat exchanger at Re of 7500 and 10000. The contours specifically focus on the midplane region and highlight the variations across different TTI designs. The velocity contours at the midplane were closely studied for different geometrical configurations, specifically focusing on the impacts of high porosity TTIs, low porosity TTIs, regular TTIs, and plain tubes at Re of 7500 and 10000. These findings are visually presented in Fig. 6(a-h), Fig. 7(a-h), and Fig. 8(a-h) and quantitatively summarized in Table 7, Table 8, and Table 9.

**Table 7:** Velocity ranges of Midplane contour for different flow conditions.

*Re*	Velocity Midplane Contour (m/s)			
	High Porosity TTI	Low Porosity TTI	Regular TTI	Plain Tube
7500	0.0–0.34	0.0–0.30	0.0–0.33	0.0–0.30
10000	0.0–0.45	0.0–0.39	0.0–0.43	0.00–0.39

**Table 8:** Velocity u ranges of Midplane contour for different flow conditions.

*Re*	Velocity u Midplane Contour (m/s)			
	High Porosity TTI	Low Porosity TTI	Regular TTI	Plain Tube
7500	−0.34–0.0	−0.008–0.008	−0.06–0.06	−0.008–0.008
10000	−0.45–0.0	−0.006–0.006	−0.08–0.07	−0.006–0.007

**Table 9:** Velocity v ranges of Midplane contour for different flow conditions.

*Re*	Velocity v Midplane Contour (m/s)			
	High Porosity TTI	Low Porosity TTI	Regular TTI	Plain Tube
7500	−0.05–0.06	−0.014–0.004	−0.08–0.07	−0.015–0.004
10000	−0.07–0.08	−0.011–0.003	−0.10–0.10	−0.012–0.003

The introduction of TTI, specifically those with high porosity, results in a significant increase in both the radial and tangential velocity components compared to a plain tube configuration. This observation highlights the positive impact of TTIs on the outcomes of the system. At a Re of 7500, the velocity contours exhibited a range of 0.0–0.34 m/s for high porosity TTIs, 0.0–0.30 m/s for low porosity TTIs, 0.0–0.33 m/s for regular TTIs, and 0.0–0.30 m/s for the plain tube. This observation indicates that all TTIs contribute to an increase in velocity components compared to a plain tube configuration. Moreover, TTIs with high porosity consistently exhibit the highest values of velocity components.

The results obtained in this study were reproduced at a Re of 10000. The velocity contours observed ranged from 0.0 to 0.45 m/s for high porosity TTIs, 0.0–0.39 m/s for low porosity TTIs, 0.0–0.43 m/s for regular TTIs, and 0.0–0.39 m/s for the plain tube. The observations consistently support the idea that the presence of TTIs leads to increased velocity components, highlighting the significant role of high porosity TTIs in causing these enhanced flow velocities.

Similar patterns can be observed in the axial velocity component (u), as depicted in Fig. 7(a-h) and Table 8. Likewise, the tangential velocity component (v) exhibits analogous trends, as shown in Fig. 8(a-h) and Table 9. The evaluations conducted provide a comprehensive analysis of the intricate influence of geometrical cuts made in TTI on the distribution of radial and tangential velocities within the heat exchanger structure. The inclusion of turbulent thermal interfaces leads to the emergence of altered flow patterns, resulting in increased fluid velocities. This effect is particularly pronounced when high porosity TTIs are utilized.

In brief, the axial velocity profile of the plain tube exhibits symmetry for all *Re*, with the highest velocity observed in the central region of the tube. Additionally, a substantial boundary layer is formed, characterized by a velocity gradient extending from the circumference of the tube towards its center. The introduction of TTI resulted in the partitioning of the fluid into two helical compartments, consequently leading to a reduction in the thickness of the boundary layer within both compartments. The axial velocity observed with geometrically cut-TTIs exhibited a relatively lower magnitude compared to the axial velocity observed with regular TTI configurations.

**Fig. 9:**
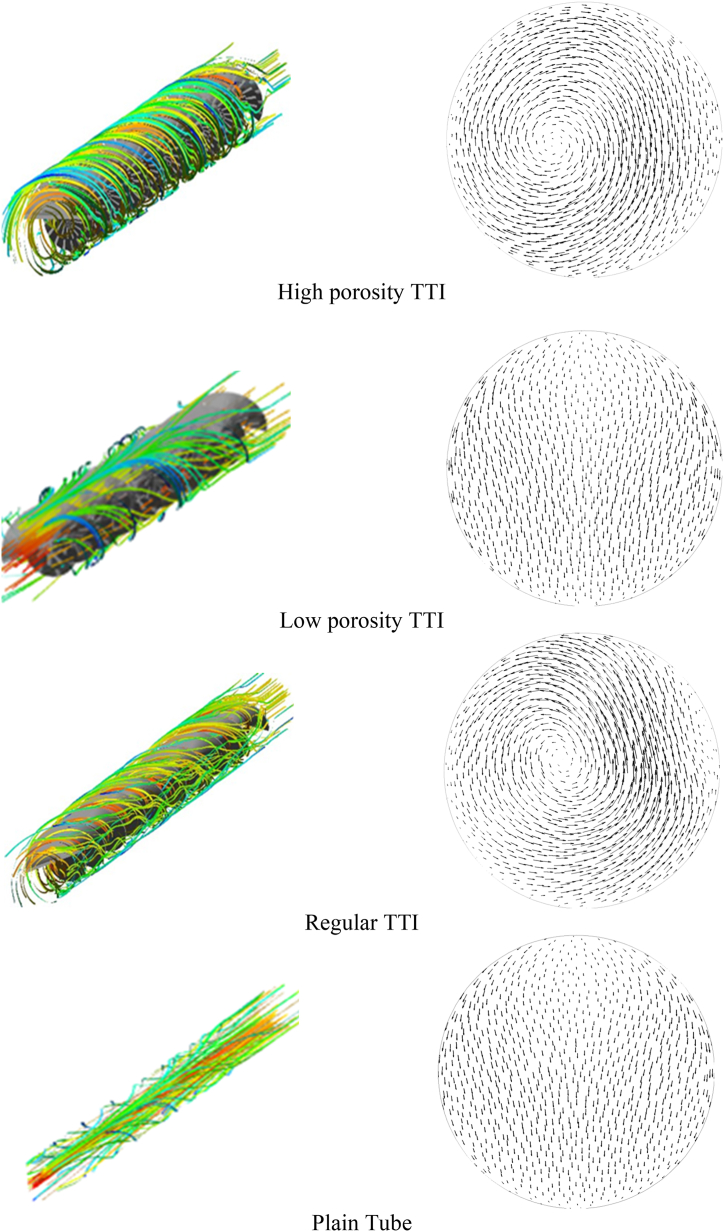
3D velocity streamline of the Inner Tube(left) and cross section velocity streamline of the outlet (right). (a) at Re 7500; flow condition: high porosity TTI, (b) at Re 7500; flow condition: low porosity TTI, (c) at Re 7500; flow condition: regular TTI, (d) at Re 7500; flow condition: plain tube.

The 3D streamlines within the double pipe heat exchanger with various geometric designs of TTIs and plain tube at Re = 7500 is shown in Fig. 9(a-d) along with the cross-sectional velocity at the outlet. It is evident that the flow pattern significantly altered after the introduction of TTI. The observed phenomenon can be ascribed to the application of geometric cuts that result in increased radial and tangential velocity. The introduction of this enhancement in the field of fluid dynamics enables more efficient mixing and enhanced heat transfer, resulting in improved performance of heat exchangers.

### 3.4 Hydrothermal performance of different inserts

**Fig. 10:**
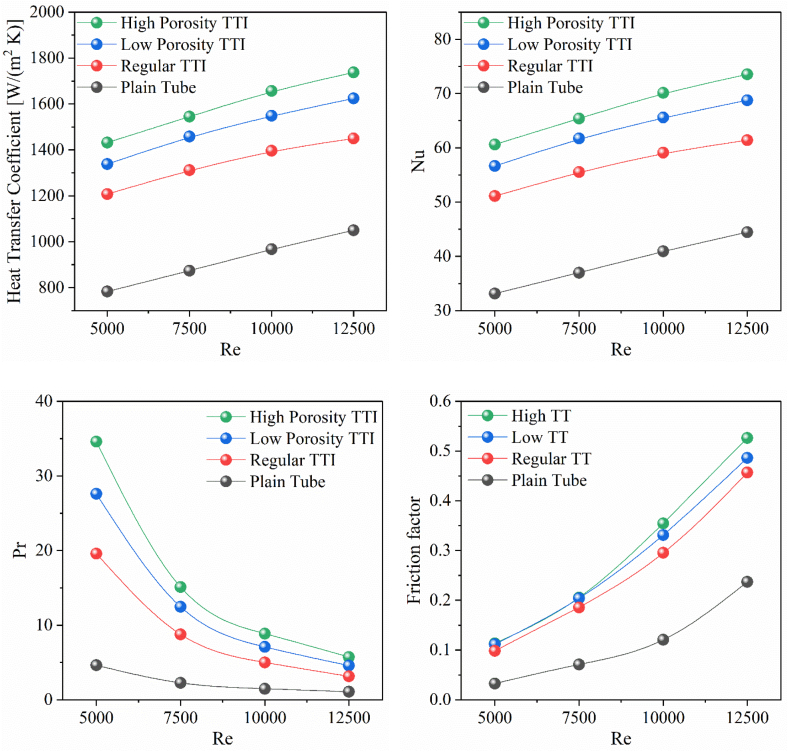
(a) Convective heat transfer coefficient vs *Re*, (b) *Nu* vs *Re*, (c) Pr vs Re and (d) friction factor vs Re for different TTIs and plain tube.

A thorough analysis of the hydrothermal performance at different *Re*, ranging from 5000 to 12500, offers significant insights into the specific effects of different insert designs in a double pipe heat exchanger. The trendlines of heat transfer coefficient (HTC) profiles, as illustrated in Fig. 10(a), demonstrates the substantial impact exerted by TTIs. It is worth mentioning that TTIs with high porosity consistently demonstrate higher HTC across a wide range of *Re*. These high porosity TTIs outperform both low porosity and regular TTIs, as well as plain tubes, in terms of heat transfer performance. This substantial improvement draws attention to the heightened capacity for heat transfer exhibited by high porosity TTIs owing to their specific geometric characteristics. These characteristics facilitate greater surface contact and fluid mixing, thereby enhancing the overall heat transfer potential.

The analysis of *Nu* trends at different *Re*, as depicted in Fig. 10(b), reveals the significant influence of insert configurations on convective heat transfer. It is noteworthy that TTIs with high porosity exhibit consistently better performance in terms of *Nu*, especially when considering lower *Re*. The aforementioned observation highlights the efficacy of high porosity designs in augmenting fluid mixing and amplifying convective heat transfer. From a quantitative standpoint, it is evident that high porosity TTIs possess a notable level of superiority. At a Re of 5000, it has been observed that high porosity TTIs exhibit a significant improvement of 82.8 % compared to plain tubes. This is followed by low porosity TTIs, which demonstrate a 70.9 % enhancement, and regular TTIs, which show a 54.2 % improvement. In a comparable manner, when considering a Re of 12500, it is observed that high porosity tube TTIs exhibit a superior performance compared to plain tubes, with an improvement of 65.4 %. Subsequently, low porosity TTIs demonstrate a performance enhancement of 54.7 %, while regular TTIs exhibit a comparatively lower improvement of 38.11 %. In addition, Fig. 10(b) investigates the relationship between HTC number and *Nu*, highlighting their interdependence. Higher HTC values are associated with increased *Nu*, which suggest enhanced convective heat transfer due to improved fluid mixing. The alignment of the inserts in this context serves to emphasize the increased efficiency of heat exchange due to the intensified fluid motion induced by the presence of the inserts.

Fig. 10(c) highlights the significance of Prandtl number (*Pr)* variations, providing a deeper understanding of fluid behavior. The inverse relationship between Pr and heat transfer efficiency becomes apparent—the lower the *Pr*, the more effective the heat transfer capacity of the fluid. This implies a heightened ability to efficiently exchange thermal energy. Notably, the observed trend aligns with the HTC and *Nu* behaviors, reinforcing the pivotal role of insert designs in influencing hydrothermal performance. These findings highlight their potential to enhance the efficiency of heat exchangers, intensify convective heat transfer, and improve overall thermal management performance. These benefits can be realized across a wide range of *Re*.

Fig. 10(d) illustrates the correlation between the average friction factor and the *Re*. The high porosity TTI consistently shows higher friction factors compared to other TTI designs, which supports the observed trend in pressure drop contours. This alignment highlights the impact of high porosity TTIs in causing flow turbulence through surface incisions.

High porosity TTIs consistently exhibit the highest friction factors, followed by low porosity TTIs and regular TTIs, when compared to the plain tube configuration, across the entire Re range. This trend visually demonstrates the impact of design variations on friction factors and pressure drop. Comparing TTIs demonstrates significant improvements in friction factor. The TTI embeds with high porosity experienced the greatest improvement, ranging from 0.113 to 0.526. The low porosity TTI embeds had a range of 0.112–0.486, while the standard TTI embeds had a range of 0.098–0.457, both compared to the plain tube configuration. The high porosity of TTIs has the ability to increase friction factors and pressure drops, which highlights their importance in improving flow turbulence and optimizing heat transfer efficiency despite its negative effect on pumping power requirements.

### 3.5 Turbulence kinetic energy

**Fig. 11:**
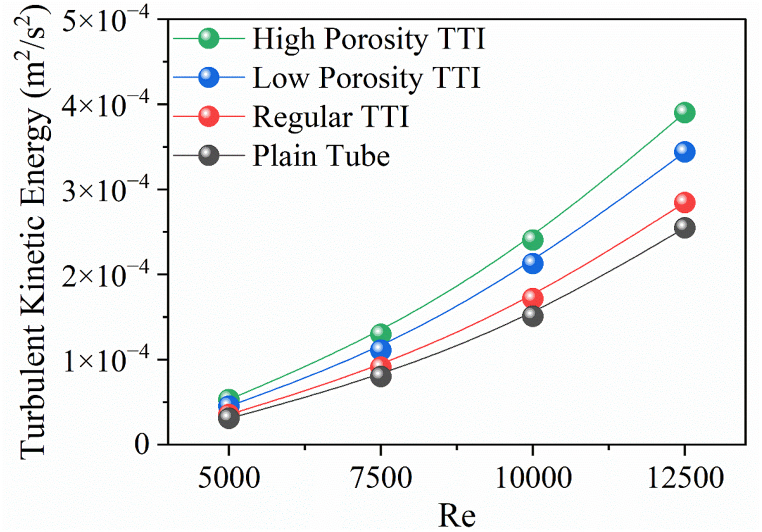
Turbulence Kinetic Energy vs Re

The turbulence kinetic energy (TKE) in the double pipe heat exchanger exhibits distinct behavior at different *Re*, which are greatly affected by various TTI configurations. As illustrated in Fig. 11, the most substantial increase in TKE occurs at a Re of 12500. TTIs with high porosity contribute to this increase, resulting in a TKE value of 0.000390 which corresponds to a 53.1 % increment compared to the plain tube configuration. The low porosity TTI shows a significant increase of 35 % in TKE, whereas the regular TTI experiences a 11.7 % increase in TKE. The relative percentage enhancement in TKE at a Re of 5000 should be taken into consideration. The high porosity TTI exhibits a significant increase of 71.56 %, highlighting its substantial impact on enhancing fluid turbulence. The improvements in low porosity and regular TTIs at a Re of 5000 are 46.7 % and 14.9 %, respectively. This further supports the significance of insert design in influencing fluid dynamics and TKE levels. This analysis highlights the ability of different TTIs, especially those with high porosity configurations, to greatly enhance fluid turbulence and improve heat transfer performance in heat exchanger systems.

### 3.6 Performance Evaluation Criteria

**Fig. 12:**
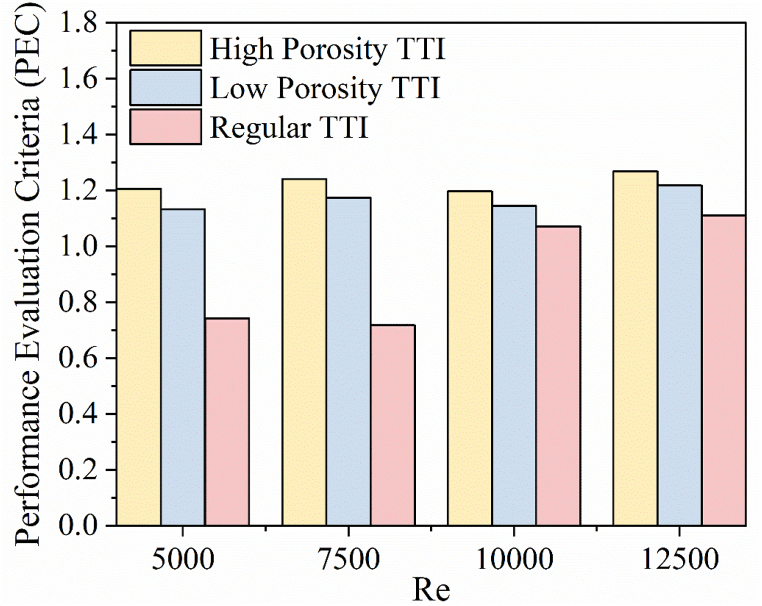
Performance Evaluation Criteria vs Re.

The Performance Evaluation Criteria (PEC) is used as a metric to measure the efficiency of different TTI configurations. Fig. 12 demonstrates that the PEC reaches its maximum value of 1.268 at a Re of 12500. The use of high porosity TTIs greatly improves the performance of the system, especially at this *Re*. Across all Re investigated, the high porosity TTI consistently exhibits superior performance in terms of PEC compared to both the low porosity TTI and the regular TTI. The PEC values at different Re highlight the differences between high and low porosity TTI. For high porosity TTI, the range is 1.206–1.268, whereas for low porosity TTI, the range is 1.133–1.217. The PEC values of the standard TTIs range from 0.742 to 1.110, in comparison to the plain tube configuration. The use of high porosity TTI in a double-pipe heat exchanger results in a significant performance improvement of over 6.44 % compared to low-porosity TTI. Moreover, high porosity TTI demonstrates a remarkable 62.5 % improvement in efficiency compared to regular TTI, emphasizing its superior performance.

The PEC analysis demonstrates that the use of high porosity TTIs greatly improves the performance of the double pipe heat exchanger system. This comparative evaluation supports the superiority of high porosity TTI in comparison to low porosity and regular alternatives. It establishes high porosity TTI as the optimal choice for enhancing heat transfer efficiency and system performance.

## 4 Summary and conclusions

This comprehensive research study aims to investigate the hydrothermal performance of different twisted tape insert designs in a double pipe heat exchanger. The findings derived from this investigation yielded a number of noteworthy outcomes:

• The utilization of porous TTI within the heat exchanger system has yielded significant effects on the distribution of temperatures. Notably, TTIs with high porosity have consistently demonstrated the ability to reduce temperature spans when compared to conventional TTI configuration. Furthermore, the incorporation of TTI, has been observed to result in amplified fluid velocities. This phenomenon serves to emphasize the significant contribution of TTIs in augmenting the overall heat transfer process.
• The experimental results consistently demonstrated that TTIs with high porosity exhibited superior performance in comparison to TTIs with low porosity, regular TTIs, and plain tubes, specifically in terms of heat transfer coefficient and *Nu.* The aforementioned findings underscore the considerable potential of high TTI in enhancing heat transfer efficiency and overall performance in thermal management.
• The analysis of turbulent kinetic energy (TKE) demonstrated that TTIs with high porosity, specifically at a Reynolds number (Re) of 12500, exhibited a significant augmentation in TKE. This observation suggests that the presence of increased fluid turbulence and enhanced heat transfer performance can be attributed to the high porosity of these TTIs. The observed effect was particularly significant for TTIs with high porosity, as they exhibited a substantial increase of 53.1 % in turbulent kinetic energy (TKE) compared to plain tubes at a Reynolds number (Re) of 12500.
• The analysis of the performance evaluation criterion data revealed that TTIs with high porosity exhibited superior performance compared to TTI with low porosity and regular TTI, across all Reynolds numbers that were investigated. The results of the study indicate that TTIs with high porosity demonstrated a significant performance enhancement of more than 6.44 % when compared to TTIs with low porosity. Furthermore, the high porosity TTIs exhibited an impressive improvement of 62.5 % in comparison to regular TTIs. These findings establish TTIs with high porosity as the preferred option for enhancing heat transfer efficiency and overall system performance.

In conclusion, this study highlights the significant impact of TTI designs on the hydrothermal performance of double pipe heat exchangers. TTIs with high porosity have been identified as a promising solution for enhancing heat transfer efficiency, fluid mixing, and thermal management. These TTIs exhibit a significant capability to improve the overall performance in these areas. The findings of this study hold significant implications for the advancement and enhancement of heat exchanger systems. These implications present prospects for increased energy efficiency and enhanced performance in a wide range of industrial settings.

## 5 Future recommendations

Future research should include economic evaluations, and a comprehensive parametric investigation. Economic analyses will be performed to determine the cost-effectiveness of porous twisted tape inserts in industrial heat exchangers, taking into account both initial expenses and long-term advantages. A comprehensive parametric study examining various Reynolds numbers, geometry, and materials will develop heat exchanger designs for improved efficiency in industrial applications.

## Data availability statement

Data will be made available on request.

## CRediT authorship contribution statement

**Md Tauhidur Rahman:** Conceptualization, Formal analysis, Methodology, Validation, Writing – original draft. **Khairul Habib:** Conceptualization, Funding acquisition, Project administration, Visualization, Writing – review & editing. **Md Niamul Quader:** Conceptualization, Data curation, Writing – original draft. **Navid Aslfattahi:** Methodology, Visualization, Writing – review & editing. **Kumaran Kadirgama:** Supervision, Visualization, Writing – review & editing. **Likhan Das:** Software, Supervision, Validation, Writing – original draft, Writing – review & editing.

## Declaration of competing interest

The authors declare that they have no known competing financial interests or personal relationships that could have appeared to influence the work reported in this paper.
